# Bacterial Microbiota of Field-Collected *Helicoverpa zea* (Lepidoptera: Noctuidae) from Transgenic Bt and Non-Bt Cotton

**DOI:** 10.3390/microorganisms9040878

**Published:** 2021-04-20

**Authors:** Jean M. Deguenon, Anirudh Dhammi, Loganathan Ponnusamy, Nicholas V. Travanty, Grayson Cave, Roger Lawrie, Dan Mott, Dominic Reisig, Ryan Kurtz, R. Michael Roe

**Affiliations:** 1 Department of Entomology and Plant Pathology, North Carolina State University, 3230 Ligon Street, Campus Box 7647, Raleigh, NC 27695-7647, USA; jdeguen@ncsu.edu (J.M.D.); adhammi@ncsu.edu (A.D.); nvtravan@ncsu.edu (N.V.T.); glcave@ncsu.edu (G.C.); rdlawrie@ncsu.edu (R.L.); dmott@ncsu.edu (D.M.); ddreisig@ncsu.edu (D.R.); michael_roe@ncsu.edu (R.M.R.); 2 Cotton Incorporated, Cary, NC 27513, USA; rkurtz@cottoninc.com

**Keywords:** *Helicoverpa zea*, cotton, Bt, resistance, tolerance, microbiota

## Abstract

The bollworm, *Helicoverpa zea* (Boddie) (Lepidoptera: Noctuidae), is an important agricultural pest in U.S. cotton and is managed using transgenic hybrids that produce insecticidal proteins from the bacterium, *Bacillus thuringiensis* (Bt). The reduced efficacy against *H. zea* caterpillars of Bt plants expressing Cry toxins is increasing in the field. In a first step towards understanding Bt cotton–bollworm–microbiota interactions, we investigated the internal bacterial microbiota of second–third stadium *H. zea* collected in the field from non-Bt versus Bt (WideStrike) cotton in close proximity (in North Carolina, USA). The bacterial populations were analyzed using culture-dependent and -independent molecular approaches. We found that WideStrike samples had a higher bacterial density and diversity per larva than insects collected from non-Bt cotton over two field seasons: 8.42 ± 0.23 and 5.36 ± 0.75 (log_10_ colony forming units per insect) for WideStrike compared to 6.82 ± 0.20 and 4.30 ± 0.56 for non-Bt cotton for seasons 1 and 2, respectively. Fifteen phyla, 103 families, and 229 genera were identified after performing Illumina sequencing of the 16S rRNA. At the family level, Enterobacteriaceae and Enterococcaceae were the most abundant taxa. The Enterococcaceae family was comprised mostly of Enterococcus species (*E. casseliflavus* and another *Enterococcus* sp.). Members of the *Enterococcus* genus can acidify their environment and can potentially reduce the alkaline activation of some Bt toxins. These findings argue for more research to better understand the role of cotton–bollworm–bacteria interactions and the impact on Bt toxin caterpillar susceptibility.

## 1. Introduction

Cotton (*Gossypium hirsutum* L.) is a fiber, feed, and food crop of global significance [1]. The U.S. is the third-largest cotton producer worldwide, with more than 10 million acres planted in 2016. As of July 2017, 85% of the U.S. cotton was transgenic [2,3], developed in the 1990s by introducing into the plant the genes for the Cry protein toxins from *Bacillus thuringiensis* (Bt) [4]. The toxins are activated in the alkaline, lepidopteran larval midgut [5]. Caterpillars in the bollworm complex including the pink bollworm *Pectinophora gossypiella* (Saunders) (Lepidoptera: Gelechiidae) and the American cotton bollworm *Helicoverpa zea* (Boddie) (Lepidoptera: Noctuidae) are important cotton pests in the USA [6].

Bt cotton has been in use for over two decades and has provided significant benefits, e.g., reducing the need for chemical insecticides. For example, the number of insecticide applications was reduced by at least 50% compared to non-transgenic cotton in Arizona [7]. Bt cotton also is harmless to non-target and beneficial invertebrates [6]. The challenge more recently, however, has been the evolution of field resistance to the Bt toxins in several caterpillar species [2,8,9,10,11].

Pest resistance to the Cry toxins is now well established [2,12]. The most tangible evidence of practical resistance, defined as field-evolved resistance that reduces pesticide efficacy to a level that has consequences for pest control [13], for *H. zea* to Cry toxins in cotton, was provided recently by Reisig et al. [2] based on long-term empirical and observational data. Understanding the mechanism(s) of Bt resistance is critical to the long-term effectiveness of Bt crops and resistance management.

In Lepidoptera in general and in *H. zea* in particular, Cry resistance has been attributed to several factors. Caccia et al. [14] detected a significant decrease in the activity of alkaline phosphatase (ALP, a Bt toxin receptor) in midgut brush border membrane vesicles (BBMVs) in laboratory-selected Cry1Ac-resistant (AR and AR1) *H. zea* larvae compared to a susceptible strain (LC). They proposed this as the mechanism of resistance to Cry1Ac because of reduced toxin binding and increased degradation by proteolysis. Zhang et al. [15] found no differences in ALP activity between their *H. zea* resistant and susceptible strains but instead came to the conclusion that a decrease in Cry1Ac activation by midgut proteases partially contributed to Cry1Ac resistance in their GA (a field-selected population) and GA-R (derived from GA and further selected in the laboratory for increased resistance) strains. Lawrie et al. [16] also suggested that an enhanced immune system may be involved, shown by the increased differential expression of three immune pathways in RNAseq comparisons of Bt-resistant versus susceptible strains of *H. zea*. RNAseq analyses also revealed several additional differences associated with resistance and included the already recognized known mechanisms of Bt resistance in the same bollworm population [16].

Another possible contributing factor to tolerance or resistance to Bt could be gut symbionts. In fact, there is evidence bacteria can affect food digestion and host nutrition, provide protection from pathogens and parasitoids, and degrade toxic compounds [17,18,19,20,21,22]. Furthermore, bacteria-mediated insect resistance to traditional chemical insecticides (organophosphates, carbamates, etc.) was suggested in Hemiptera [23], Diptera [24], and Lepidoptera [25,26,27]. For example, Kikuchi et al. [23] demonstrated that *Burkholderia* confers insecticide resistance in the bean bug, *Riptortus pedestris* (Fabricius) (Hemiptera: Alydidae), to fenitrothion, a widely used organophosphate. Cheng et al. [24] showed that the gut symbiont, *Citrobacter* sp. (CF-BD), isolated from the oriental fruit fly, *Bactrocera dorsalis*, conferred resistance to trichlorphon by increased degradation. When it comes to the relationship between Bt and the insect gut microbiota, the majority of studies have focused on the mode of action and whether the septicemia caused by enteric bacteria is mandatory [28,29] or not [30,31] for Bt toxicity.

Despite the growing interest in studying the interactions between gut microbes and insecticides, little is known about the microbial communities associated with *H. zea* in cotton fields. Wang et al. [32] studied the microbiota of field-collected *H. zea* larvae but the insects were collected from sweet corn. Additionally, they used culture-dependent techniques and focused only on bacteria found in fresh oral secretions. The aim of the current study was to determine the bacterial community composition of cotton bollworms from insects (2nd to 3rd stadium) collected from non-Bt versus Bt (WideStrike) cotton grown in the same field in North Carolina. Cultivable bacteria were enumerated, and the total *H. zea*-associated bacterial community structure was analyzed using culture-independent DGGE and next-generation sequencing of 16S rRNA gene amplicons.

## 2. Materials and Methods

### 2.1. Season 1

#### 2.1.1. Insect Collection, Identification and Sample Preparation

**Figure 1:**
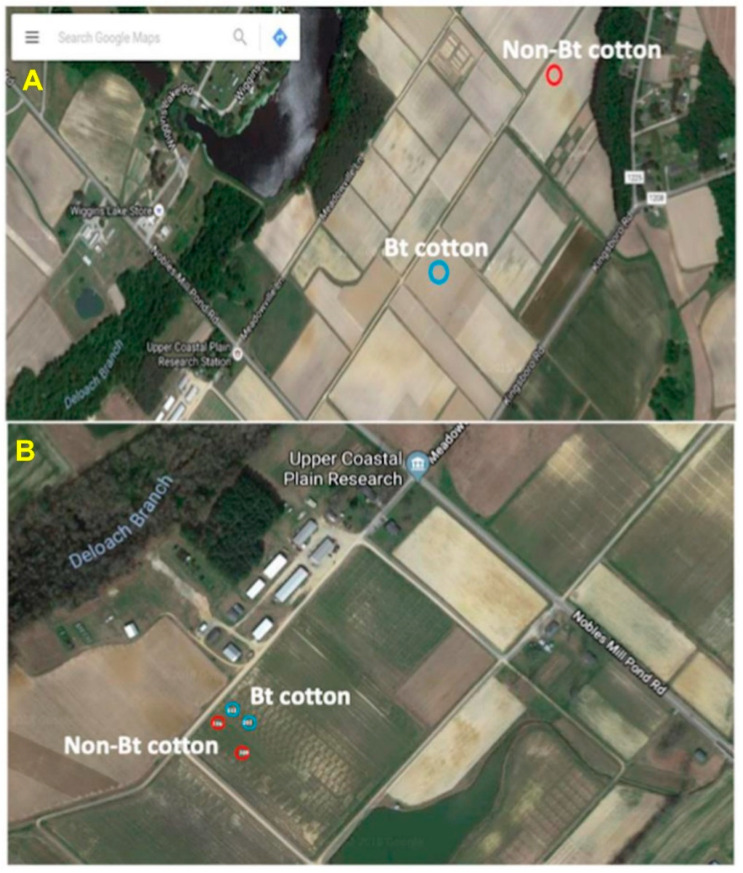
*Helicoverpa zea* collection sites at the Upper Coastal Plain Research Station, Rocky Mount, NC (USA). (**A**) Larvae were collected from two adjacent fields in the fall of 2016 from non-Bt and Bt (WideStrike, Cry1Ac + Cry1F) cotton; (**B**) larvae were collected from adjacent plots in the same field as indicated in the figure in the fall of 2018.

*Helicoverpa zea* larvae were collected in August 2016 from non-Bt (PHY425RF) and Bt (WideStrike, PHY499WRF, Cry1Ac + Cry1F) (Corteva, Wilmington, DE, USA) cotton fields located at the Upper Coastal Plain Research Station, Rocky Mount, NC, USA. The locations of the collections are shown in Figure 1A. The presence of larvae on the plants (squares and bolls) was checked, and the plant structures containing the larvae were removed and placed individually in 29.6 mL cups with lids (Solo Cup Company, Highland Park, IL, USA). The cups were placed on ice and the collections were transferred to the laboratory and processed the same day. The bollworm stadium and species identifications were based on morphological characteristics [33,34,35] and second and third stadium larvae were used for the studies.

#### 2.1.2. Quantification of Cultivable Bacteria

The number of cultivable bacteria per caterpillar from non-Bt and Bt cotton was estimated using a Tryptic Soy Agar (TSA) medium (Diftco^TM^, Becton, Dickson and Company, Sparks, MD, USA). Fourteen larvae (7 per treatment) were separately surface sterilized with 95% ethanol (30 s) followed by 1% bleach (30 s) and finally, washed 5 times with sterile water. The final washes were pooled together per treatment for subsequent verification of sterility through bacterial culture. The larvae were transferred to sterile 2 mL microcentrifuge tubes (Fisher Scientific, Pittsburgh, PA, USA), each containing 10 sterilized 3 mm solid glass beads (Fisher Scientific, Pittsburgh, PA, USA) and were homogenized in 300 µL of sterile phosphate-buffered saline (PBS, pH = 7.4) (Life Technologies Corp., Frederick, MD, USA) in the FastPrep™ FP120 system (Thermo Electron Corporation, Waltham, MA, USA) for 45 s. To enumerate total cultivable bacteria, homogenate from each sample was serially diluted up to 10^−7^ and then drop plated (25 μL/each dilution) on the surface of the medium using the drop plating method [36,37] and incubated at 28 °C for 24 h. Colonies were counted and colony forming units (CFUs)/insect calculated using dilutions with the maximum CFUs (6 to 60 colonies) [36]. Statistical differences in total number of cultivable bacteria (log-converted) between larvae collected from Bt versus non-Bt cotton were estimated with a Mann–Whitney *U*-test using the R statistical software [38].

#### 2.1.3. Denaturing Gradient Gel Electrophoresis

Denaturing gradient gel electrophoresis (DGGE) was performed to investigate the bacterial community diversity of the samples collected during season 1. Total DNA was extracted from three samples per treatment using the method previously described by Ponnusamy et al. [39]. Briefly, 200 µL of homogenate (described earlier) were transferred to a 1.5 mL Eppendorf tube containing 160 µL of lysis buffer 1 (with 20 µL of lysozyme and 20 µL of proteinase K). The samples were then incubated at 37 °C for 1 h. Subsequently, 200 µL of pre-warmed lysis buffer 2 were added with further incubation at 56 °C for 1 h. DNA was recovered through phenol/chloroform extraction and ethanol precipitation, and the resulting DNA pellet was resuspended in 100 µL of nuclease-free water (Life Technologies Corp., Frederick, MD, USA). Subsequently, the DNA was further purified with the WIZARD DNA Cleanup System (Promega, Madison, WI, USA). The V3 region of the 16S rRNA gene was then amplified using the universal primer F357-GC (5′-GC-clamp+ CCTACGGGAGGCAGCAG-3′) and 518R (5′-ATTACCGCGGCTGCTGG-3′) with a GC-clamp added to the 5′ end of the forward primer to prevent complete denaturation of the double-stranded fragments. Touchdown PCR and DGGE gel electrophoresis were performed as described by Ponnusamy et al. [40].

### 2.2. Season 2

Since differences were found in season 1 both in cultivable bacteria per insect and bacterial diversity between treatments (see Results and Discussion for more detail), this justified repeating the research in season 2 and using a higher resolution method to determine bacteria diversity.

#### 2.2.1. Insect Collection

Twenty-two third stadium larvae of *H. zea* were collected in August 2018 from non-Bt (n = 7, PHY425RF; we could not find more larvae in the plots at the time of collection) and Bt (n = 15, PHY444WRF, Cry1Ac + Cry1F) cotton (Corteva, Wilmington, DE, USA) located at the Upper Coastal Plain Research Station, Rocky Mount, NC, USA from adjacent field plots (Figure 1B). Insect collections, as well as stadium and species identifications, were performed as described earlier.

#### 2.2.2. Quantification of Cultivable Bacteria

Larvae were separately surface sterilized the same day they were transferred to the laboratory and homogenized in sterile PBS as described in season 1. Homogenates were then used subsequently for quantification of cultivable bacteria and DNA extraction. For cultivable bacteria, homogenates from the different samples were drop plated on the surface of Petri dishes (as described earlier) containing Plate Count Agar (PCA, Diftco^TM^, Becton, Dickson and Company, Sparks, MD, USA), commonly used to assess total cultivable heterotrophic bacterial growth.

#### 2.2.3. DNA Extraction

DNA extractions were performed using a DNeasy Blood and Tissue extraction kit (Qiagen, Hilden, Germany) per the manufacturer’s protocol. Two hundred microliters of each larval homogenate were used, and DNA was eluted in 60 µL of elution buffer (AE buffer). The DNA samples were further purified using the Wizard DNA cleanup system (Promega, Madison, WI, USA). DNA quality and quantity were assessed using a NanoDrop 1000 spectrophotometer (Thermo Fisher Scientific, Waltham, MA, USA). The genomic DNA samples were normalized to 50–100 ng/µL and stored at −40 °C until further use.

#### 2.2.4. NGS Library Preparation and Sequencing

Bacterial 16 rRNA gene fragments were amplified using universal V3–V4 hypervariable region-specific primers [41]. Amplification was performed with: forward primer (Illumina adapter+341F) and reverse primer (Illumina adapter+806R). The 16S rRNA sequencing libraries were constructed according to Illumina’s 16S rRNA metagenomics sequencing library preparation protocol (Illumina, San Diego, CA, USA). Amplicon products were verified on a 1.5% agarose gel. Libraries constructed from 21 samples (15 Bt and 6 conventional; one non-Bt sample was lost during preparation) were quantified with Quant-iT PicoGreen (Molecular Probes, Inc., Eugene, OR, USA), normalized, and then pooled prior to sequencing. Library sequencing was performed at the Microbiome Core Facility, School of Medicine, University of North Carolina, Chapel Hill, NC, USA.

#### 2.2.5. Bioinformatics

Quality filtering and analyses of the 16S rRNA gene sequence data (see Supplementary S1 File) were performed with QIIME2-2020.6 [42]. Paired-end V3-V4 sequence (2 × 300 bp) reads were demultiplexed and quality filtered using the DADA2 [43] algorithm as a QIIME2 plugin. This process joined the paired-end reads together, denoised the data, and checked and removed chimera, resulting in the identification of the amplicon sequence variants (ASVs) which are equivalent to the 100% OTUs [44,45]. Primers sequences were trimmed off the reads, and the sequences were truncated at the 230-nucleotide position to increase the quality of the reads subsequently processed. ASVs were assigned a taxonomical classification using a pretrained Naïve Bayes classifier which was trained on the Greengenes database (version 13_8 99% OTUs) [46] using the QIIME feature-classifier and classify-sklearn command. Sequence variants annotated as Mitochondria and Chloroplast were removed from the dataset and were not used for diversity analyses. For computing diversity metrics, representative sequences were aligned and masked to remove gaps, and a mid-point rooted phylogenetic tree was constructed using the QIIME2 FastTree plugin [47]. To ensure an even sampling depth, each sample was rarefied to a depth of 5000 sequences per sample. Alpha diversity was estimated with two indices: the Shannon index [48] and the number of observed OTUs (ASVs). The principal coordinate analysis (PCoA, weighted Unifrac) result was visualized using EMPEROR [49].

#### 2.2.6. Statistical Analyses

Statistical analyses for alpha and beta diversities were conducted in QIIME2. The statistical significance of alpha diversity between groups (Bt and non-Bt) was inferred using pairwise Kruskal–Wallis H-tests. To estimate β-diversity between the two groups (Bt and non-Bt), we used the weighted Unifrac distance metric [50]. For beta diversity, a PERMANOVA test was run to determine if there was any difference between the two treatments. For a given bacterial taxa of interest, the statistical difference between the two groups (Bt and non-Bt) was determined with a Mann–Whitney *U*-test using the R statistical software.

## 3. Results

### 3.1. Cultivable Bacteria: Non-Bt versus Bt Cotton

**Figure 2:**
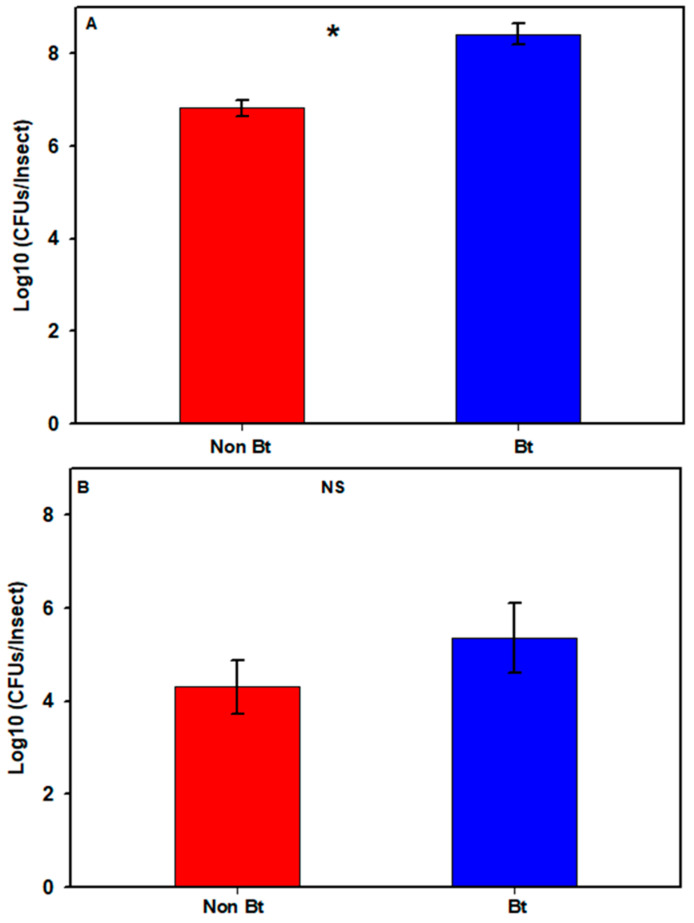
Cultivable bacterial loads in *H. zea* larvae from non-Bt cotton versus Bt cotton (WideStrike) from the field. (**A**) 2nd to 3rd stadium larvae collected in August 2016; (**B**) 3rd stadium larvae collected in August 2018. Each bar represents the mean number of Colony Forming Units + SE. Presence of an asterisk indicates significant difference between the two groups (*p* < 0.05; Mann–Whitney *U*-test); NS = non-significant difference (*p* > 0.05; Mann–Whitney *U*-test).

In the first season, the estimated total number of cultivable bacteria in the bollworms varied between 6 and 9 log_10_ CFUs/insect (see Figure S1). One non-Bt sample produced no cultivable bacteria and was considered as a technical error and was removed from the analysis (see Figure S1). Bollworms collected in that season from WideStrike (Bt) had a mean bacterial count ± SE (standard error) of 8.42 ± 0.23, compared to 6.82 ± 0.20 (log_10_ CFUs/insect) for larvae collected in non-Bt cotton (Figure 2A; see Figure S1,) with a significant difference (Mann–Whitney *U*-test, *W* = 1, *p* = 0.005). In the second season, the bacterial count in the larvae varied between 2.78 and 8.1 log_10_ CFUs/insect (see Figure S1) with means of 5.36 ± 0.75 (Bt) and 4.30 ± 0.56 (non-Bt) (Figure 2B; see Figure S1) (Mann–Whitney *U*-test, *W* = 18, *p* = 0.456).

### 3.2. DGGE Analysis: Non-Bt versus Bt Cotton

**Figure 3:**
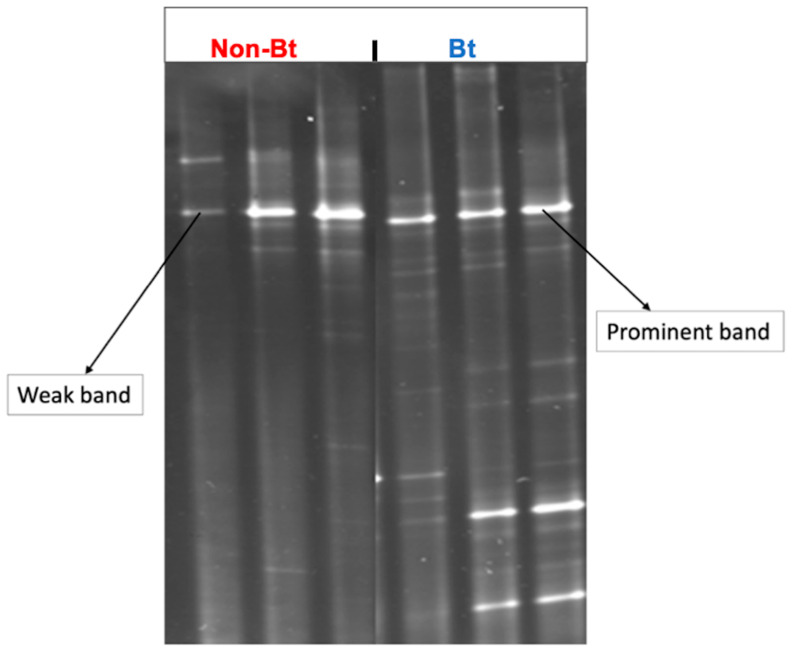
DGGE profiles of bacterial communities from the bollworm, *H. zea*, collected at the Upper Coastal Plain Research Station, Rocky Mount, NC (USA) in August 2016. Larvae were collected from non-Bt (PHY425RF) and Bt (PHY499WRF, Cry1Ac + Cry1F) cotton and processed the same day after transfer to the laboratory. DGGE was performed after the amplification of the bacterial V3 region of the 16S rRNA gene.

DGGE was conducted using the 16S rRNA gene to obtain a coarse-scale analysis of the bacterial community diversity (species richness + evenness) of the bollworms collected from Bt versus non-Bt cotton in season 1. Each band shown on the gel image (Figure 3) represents at least one unique phylotype (OTU = operational taxonomic unit = bacterial species). The number of DGGE-DNA bands reflects the species richness in a sample while the intensity of the band in DGGE relates semi-quantitatively to species abundance [51]. The results confirmed the trends observed during bacterial culturing and provided additional information about bacterial diversity in the samples. Bollworms collected from non-Bt cotton had 1–2 prominent bands and 4–6 weak bands (Figure 3) while bollworms from Bt had 2–3 prominent bands with 10–12 weak bands (Figure 3). These results suggest that the insects from the two treatments had different microbial diversity (richness + abundance) profiles.

### 3.3. Overall Structure of Bacterial Communities Associated with Bollworms in Season 2

After quality filtering, low-sequence samples were removed from the data and not processed during the subsequent analyses. These samples were defined as samples with <5000 sequence reads. Thus, a total of 1,667,663 16S rRNA gene sequences were obtained from 18 16S rRNA gene libraries (6 for non-Bt and 12 for Bt) after quality filtering using DADA2 with an average of 92,648 reads. The reads obtained after quality filtering were clustered into 2301 sequence variants and assigned to 15 phyla, 103 families, and 229 genera.

### 3.4. Alpha and Beta Diversity

**Figure 4:**
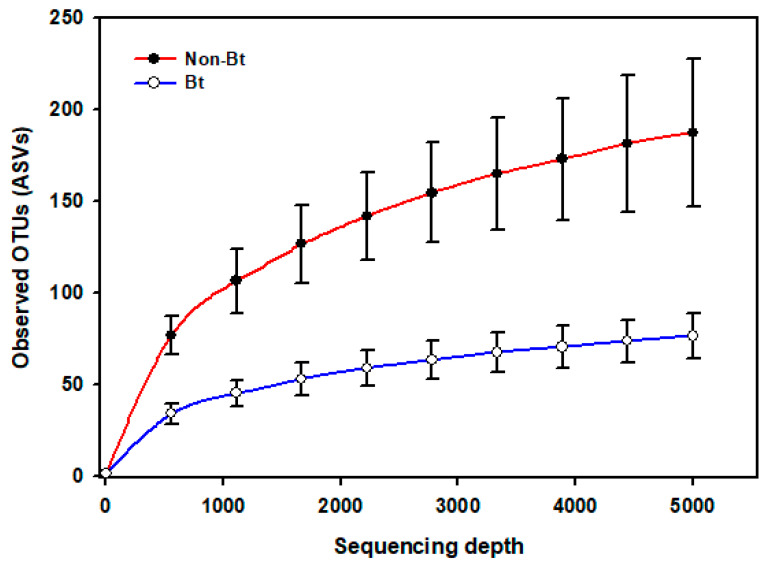
Rarefaction curves of the mean number of observed OTUs (sequence variants) from internal DNA samples of the bollworm, *Helicoverpa zea*. Samples were third stadium larvae from non-Bt (PHY425RF) and Bt (WideStrike, PHY444WRF, Cry1Ac + Cry1F) cotton in season 2. Sequences were obtained from amplified DNA fragments from V3–V4 hypervariable regions of the 16S rRNA gene.

**Figure 5:**
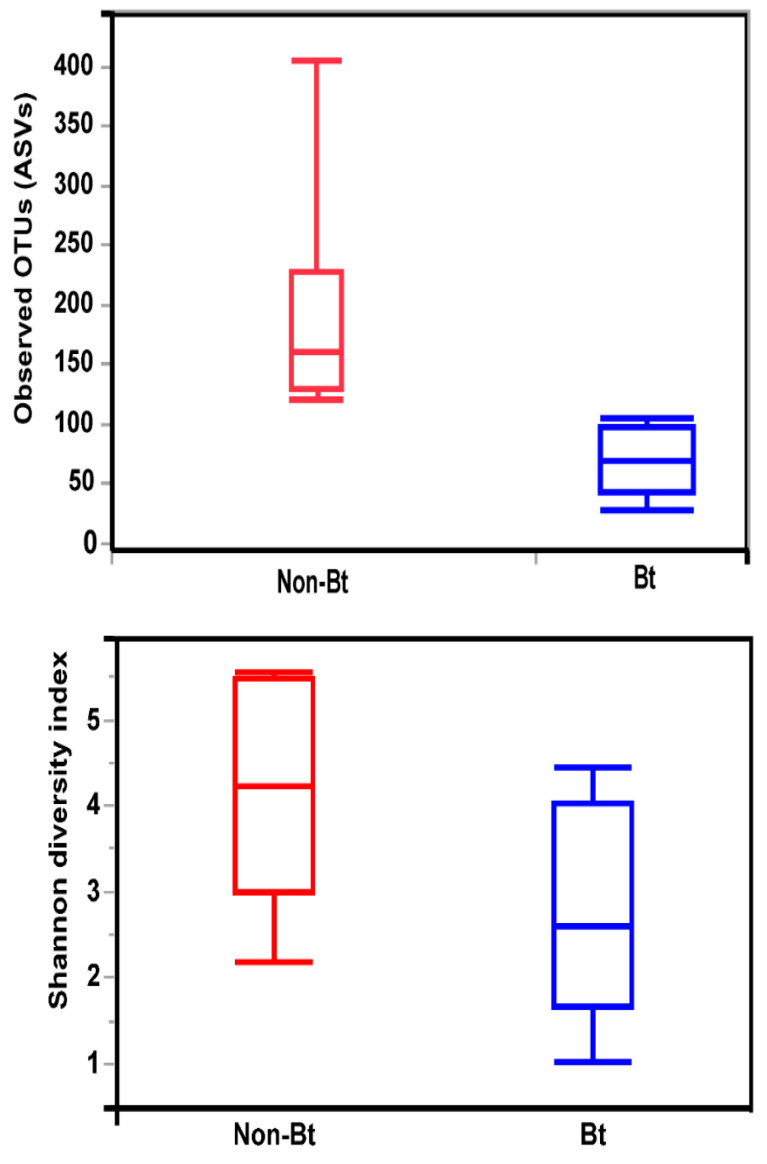
Alpha diversity measures (Observed OTUs and Shannon diversity) of the internal microbiota of third stadium larvae of the bollworm, *H. zea*. Larvae were collected from non-Bt versus Bt cotton (WideStrike) in August 2018 (field season 2).

**Figure 6:**
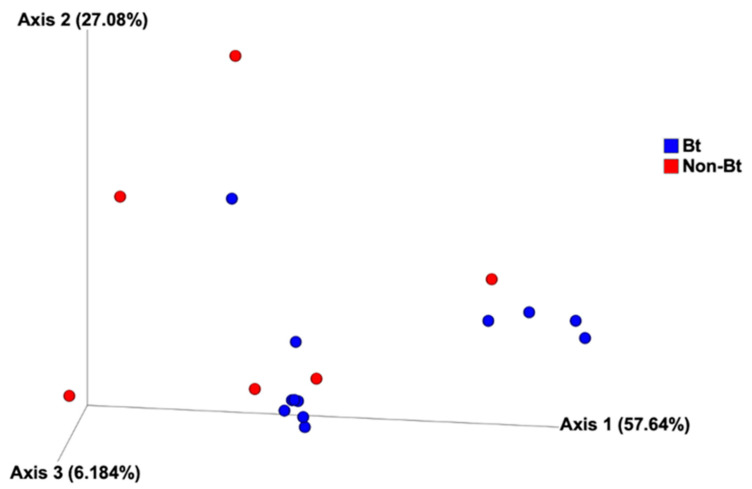
Principal coordinate analysis of bacterial composition of third stadium bollworms *Helicoverpa zea* collected from non-Bt cotton and Bt (WideStrike) in season 2. Analysis is based on the weighted Unifrac metric.

The shape of the rarefaction curves based on the observed OTUs (ASVs) suggests that the sequencing depth of 5000 used in this study allowed us to capture the majority of taxa present in the bollworm samples (Figure 4; see Figure S2). Alpha diversity analyses revealed that third stadium *H. zea* larvae collected from non-Bt cotton harbored more ASVs (OTUs) compared to Bt (WideStrike) samples (Kruskal–Wallis, *p* = 0.004) (Figure 5). However, no significant differences were noted in the Shannon diversity index between the treatments (Kruskal–Wallis, *p* = 0.05) (Figure 5). β-diversity analyses showed that the majority of the variation in bacterial diversity across samples could be explained by the origin of the samples (Figure 6). No distinct clustering of the samples per treatment group was observed (Figure 6). This was further shown by a permutation multivariate analysis of variance which was not significant (PERMANOVA, *p* = 0.07).

### 3.5. Microbial Community Composition

**Figure 7:**
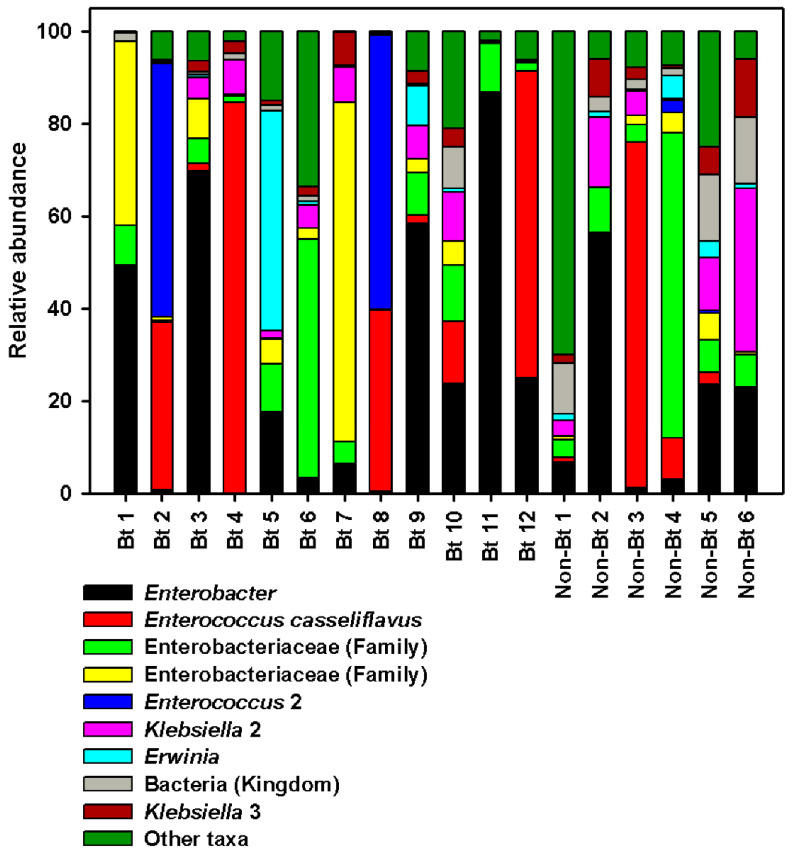
Relative abundance of major bacteria in DNA samples from *H. zea* larvae (third stadium) at the highest taxonomic resolution (level 7, species level). Bars represent proportions of each taxa. “Other taxa” refers to all the taxa with relative abundance below 3% over the total number of reads.

Overall, the most sequence-abundant bacterial taxa (abundance ≥3% across samples) at the phylum level were Proteobacteria (67.80%) and Firmicutes (27.26%) (see Supplementary S2 File). We found two families to be the most abundant across all samples: Enterobacteriaceae and Enterococcaceae. Enterobacteriaceae accounted for ~60% of the reads in both groups (non-Bt cotton versus Bt) (see Supplementary S2 File). The largest difference between the two groups was observed with Enterococcaceae even though no statistical difference was obtained (Mann–Whitney *U*-test, *p* = 0.553). In fact, 30% of the reads in the WideStrike (Bt) samples belonged to Enterococcaceae compared to 15% of the reads in non-Bt cotton (a 2-fold difference, see Supplementary S2 File). At the highest taxonomic resolution (species level), most taxa were classified up to the genus level (Figure 7; see Supplementary S2 File). At this level, four genera were identified as the most sequence-rich taxa: *Enterococcus* (with two species), *Klebsiella* (with three species), *Enterobacter,* and *Erwinia* (Figure 7; see Supplementary S2 File). *Enterococcus casseliflavus* (20% in Bt versus 15% in non-Bt) and a second non-identified *Enterococcus* species (9.5% in Bt versus 0.58% in non-Bt) were more abundant in WideStrike samples. *Klebsiella oxytoca* (the most abundant *Klebsiella* species identified) was also highly present in WideStrike samples (12% of the reads vs. 2% for non-Bt cotton). *Enterobacter* was the most abundant genus detected in our samples and comprised 19% of the reads in non-Bt cotton and 28% of the reads in WideStrike (Bt) (Figure 7; see Supplementary S2 File). Four percent of the reads (across all samples) were only classified to the Kingdom level as “bacteria” (Figure 7; see Supplementary S2 File).

## 4. Discussion

In this study, we investigated the density and diversity of bacterial communities associated with the internal body of second and third stadium larvae of the American cotton bollworm, *H. zea*, as impacted by the cotton variety. Significantly higher numbers of bacteria were cultured from bollworms collected from Bt (WideStrike) compared to those from non-Bt cotton (Figure 2A) during the first season. In the second season, even though there was no statistically significant difference, the same trend was also observed (1.2-fold difference, Figure 2B) using a different culture medium. It could be argued that, because we did not differentially culture the internal bacteria (gut versus hemocoel) from the insects, it is possible that the bollworms from WideStrike were at the septicemia stage after feeding on Cry proteins, causing the higher bacterial density and diversity. Another reasonable assumption could be that the differences found in this study are simply due to differences in the two plant varieties and unrelated to the presence and absence of Bt. However, similar results were found to ours in a previous study where resistant tobacco budworms had a higher bacterial density and diversity when fed on Bt cotton compared to non-Bt cotton [52]. Using culture and DGGE analyses, Gracy et al. [53] also found that *H. armigera* larvae collected from Bt cotton had a higher bacterial diversity compared to larvae from other plants (Pigeonpea, Chickpea and Tomato) and were also the most resistant to various insecticides (spinosad, emamectin benzoate, cypermethrin, thiodicarb). It is well-known that diet is an important factor in shaping an insect’s microbiome [20], and therefore, it is not surprising that insects feeding on non-Bt versus Bt cotton have differences in their microbiota. We do not know whether the higher bacterial load observed in larvae from Bt cotton in our study is a cause or consequence from feeding on Bt and whether this has a functional advantage for the insect, especially regarding their susceptibility to Bt. Clearly, more work needs to be conducted to investigate this.

In the first year, we used DGGE to quantify the number of DNA bands and band relative intensities to estimate overall OTU diversity. In the second year, we used high-throughput 16S rRNA gene amplicon sequencing which has a much greater resolution in measuring bacteria diversity than DGGE. The sequencing results showed that samples from non-Bt cotton harbored a significantly higher number of OTUs, but the Shannon diversity analysis revealed no significant difference between the two treatments. The principal coordinates analysis (PCoA) plot revealed no distinct clustering of WideStrike and non-Bt cotton collected caterpillars, confirmed by the PERMANOVA test, which was not significant. Even though these observations could, at first, suggest that the bacterial communities present in the two treatments are the same, the presence of several taxa that are selectively more abundant in one treatment over the other is worth noticing and examining. This is discussed in more detail later.

Overall, Proteobacteria and Firmicutes were the predominant phyla in our samples. Proteobacteria accounted for about 68% of the reads. This result is not surprising and is in agreement with findings in other insects, including Lepidoptera [32,53,54]. Two families, Enterobacteriaceae and Enterococcaceae, were detected as the most sequence-rich taxa and are commonly and consistently present in insects’ gut, especially in Lepidoptera. Enterobacteriaceae are highly abundant in Lepidoptera. Paniagua et al. [54] studied the bacterial symbionts in Lepidoptera using 30 different species and found that over 80% of the species targeted harbored members of this family. Additionally, the high abundance of this family in our samples (~60% in average in each group of samples) (see Supplementary S2 file) indicates that members of this family may be functionally important in the bollworm. Due to the high carbon-to-nitrogen ratio in plant tissues, it is probable that chewing insects such as caterpillars need to deal with limited dietary nitrogen. Enterobacteriaceae members such as *Klebsiella oxytoca* and *Enterobacter* spp., highly present in our samples, are diazotrophic bacteria and are known to help insects fix and use nitrogen [54,55]. In addition, *Enterobacter* spp. have been shown to promote herbivory on chemically defended plants likely because of their ability to detoxify plant xenobiotics [56]. Whether the bacteria mentioned above play the same roles in the *H. zea* is yet to be demonstrated.

The most striking observation in our data is the differential abundance of Enterococcaceae between Bt (WideStrike) and non-Bt cotton. Thirty percent (30%) of the reads in WideStrike belonged to this family, essentially composed of two *Enterococci* (*Enterococcus casseliflavus* and another *Enterococcus* sp.). *Enterococci* are commonly found in the gut of larval Lepidoptera, and *E. casseliflavus* has been isolated from other phytophagous lepidopterans such as *Manduca sexta* [57], *Spodoptera litura* [58], *Hyles euphorbiae,* and *Brithys crini* [19]. The guts of *H. euphorbia* and *B. crini* (two polyphagous insects that feed exclusively on latex-rich and alkaloid-rich plants) were dominated by *E. casseliflavus*, suggesting that this bacterium is involved in the tolerance developed by these insects to the toxic compounds found in their host plants. It has also been found to be associated with *Spodoptera litura* feeding on lima beans which are rich in toxic terpenes [19]. *Enterococcus* sp. were found to enhance resistance in the diamondback moth, *Plutella xylostella* [27], against the widely used insecticide chlorpyriphos. Another important characteristic of Enterococci that may be relevant here is the ability of some members of this genus (such as *E. faecalis*) to acidify their local environment [19,59]. The presence of such bacteria in *H. zea* larval guts might protect the insect against the insecticidal activity of Bt toxins which require an alkaline environment to be processed. The presence of these bacteria might result in Bt toxin tolerance or resistance (for the latter, if there is an insect genetic mechanism that transfers the bacteria from one generation to the next). Moreover, some studies have previously suggested that gut bacteria have the potential to inhibit Bt growth (not applicable in our studies) and degrade Bt toxins [60,61]. At this juncture, we cannot confirm whether the *Enterococci* highly present in the WideStrike samples in this study play a role in changing the caterpillar’s susceptibility to Bt. Additional studies are needed to clarify the role of *H. zea* gut bacteria in Bt susceptibility, especially given the fact that high levels of resistance to Bt have been found in cotton fields in North Carolina where the insects were collected [2].

## 5. Conclusions

In summary, we characterized for the first time the microbiota associated with field-collected (Bt and non-Bt cotton) second and third stadium cotton bollworms, *H. zea*, using culture techniques, DGGE and Illumina MiSeq next-generation sequencing (NGS). We found higher levels of internal cultivable bacteria in bollworms from WideStrike in seasons 1 and 2 but the differences were only statistically significant for season 1. The results also showed that a few taxa dominated the microbiota of the caterpillars at each taxonomical classification level. The most intriguing result was the presence, in high abundances, of Enterococcaceae (essentially *Enterococci*), especially in Bt (WideStrike) samples. *Enterococcus* spp. have been shown to enhance resistance to conventional insecticides and some members of this genus can acidify their environment, which could increase tolerance towards Bt by decreasing its activation. Therefore, studies with larger sample sizes and with varying collection sites should be conducted to characterize the role that these bacteria may play in *H. zea* larvae feeding on Bt cotton.

## Data Availability

The NGS data presented in this study are openly available in the NCBI GenBank under SRA accession: PRJNA719357.

## Supplementary Materials

The following are available online at https://www.mdpi.com/article/10.3390/microorganisms9040878/s1, Figure S1: Detailed prevalence (per sample) of cultivable bacterial loads in bollworms from non-Bt cotton versus Bt (WideStrike) cotton from the field. (A) 2nd to 3rd stadium *H. zea* larvae collected in August 2016; (B) 3rd stadium larvae collected in August 2018. Each bar represents the median number (log-converted) of Colony Forming Units; Figure S2: Rarefaction curves of the number of observed OTUs (sequence variants) from internal DNA samples of the cotton bollworm, *H. zea*. Bt: sample originating from third-stadium larvae from Bt (WideStrike, Cry1Ac + Cry1F) cotton. Non-Bt: sample originating from third-stadium larvae from non-Bt cotton. Number after sample origin represents sample number. Sequences were obtained from amplified DNA fragments overlapping the V3–V4 hypervariable regions of the 16S rRNA gene; Supplementary S1 File: Mapping file; Supplementary S2 File: Relative abundance of major bacteria in DNA samples from field-collected *H. zea* larvae (3rd stadium) at phylum, family, genus and species level.
