# Supplementary material for: Bacterial Microbiota of Field-Collected Helicoverpa zea (Lepidoptera: Noctuidae) from Transgenic Bt and Non-Bt Cotton
Source: Microorganisms. 2021 Apr 20;9(4):878. doi: 10.3390/microorganisms9040878 (PMC8072973; doi:10.3390/microorganisms9040878)
Supplement: Supplementary file 1 [file microorganisms-09-00878-s001.zip › microorganisms-1172465-supplementary/microorganisms-1172465-supplementary.pdf]

Bacterial Microbiota of Field-Collected *Helicoverpa zea* (Lepidoptera: Noctuidae) from Transgenic Bt and Non-Bt Cotton

Jean M. Deguenon [1], Anirudh Dhammi [1], Loganathan Ponnusamy [1,*], Nicholas V. Travanty [1], Grayson Cave [1], Roger Lawrie [1], Dan Mott [1], Dominic Reisig [1], Ryan Kurtz [2] and R. Michael Roe [1]

[1] Department of Entomology and Plant Pathology, 3230 Ligon Street, Campus Box 7647, North Carolina State University, Raleigh, NC 27695-7647, USA; jdeguen@ncsu.edu (J.M.D.); adhammi@ncsu.edu (A.D.); nvtravan@ncsu.edu (N.V.T.); glcave@ncsu.edu (G.C.); rdlawrie@ncsu.edu (R.L.); dmott@ncsu.edu (D.M.); ddreisig@ncsu.edu (D.R.); michael_roe@ncsu.edu (R.M.R.)

[2] Cotton Incorporated, Cary, NC 27513, USA; rkurtz@cottoninc.com

* Correspondence: lponnus@ncsu.edu

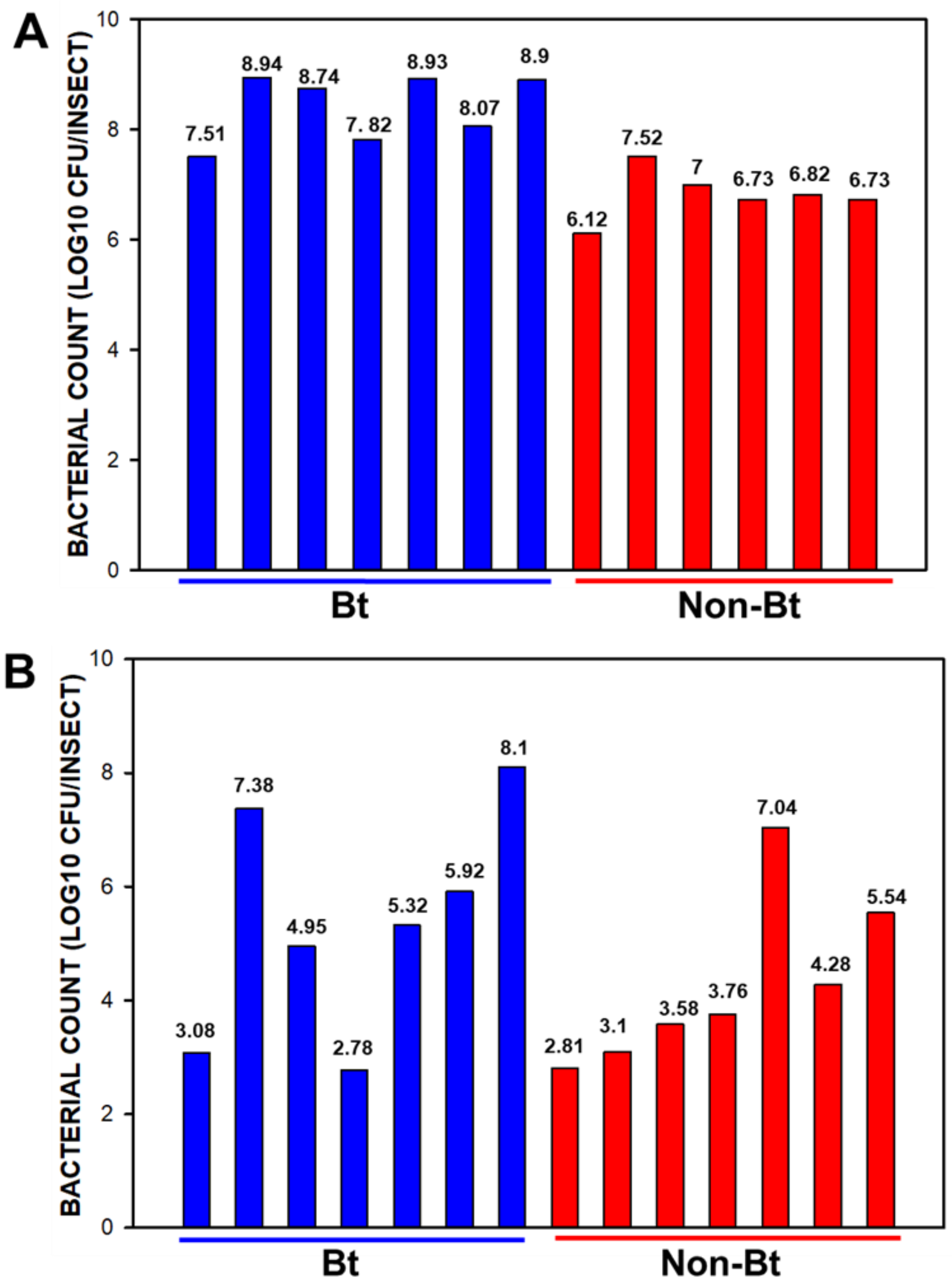

Figure S1. Detailed prevalence (per sample) of cultivable bacterial loads in bollworms from non-Bt cotton versus Bt (WideStrike) cotton from the field. (A) 2nd to 3rd stadium *Helicoverpa zea* larvae collected in August 2016; (B) 3rd stadium bollworms collected in August 2018. Each bar represents the median number (log-converted) of Colony Forming Units.

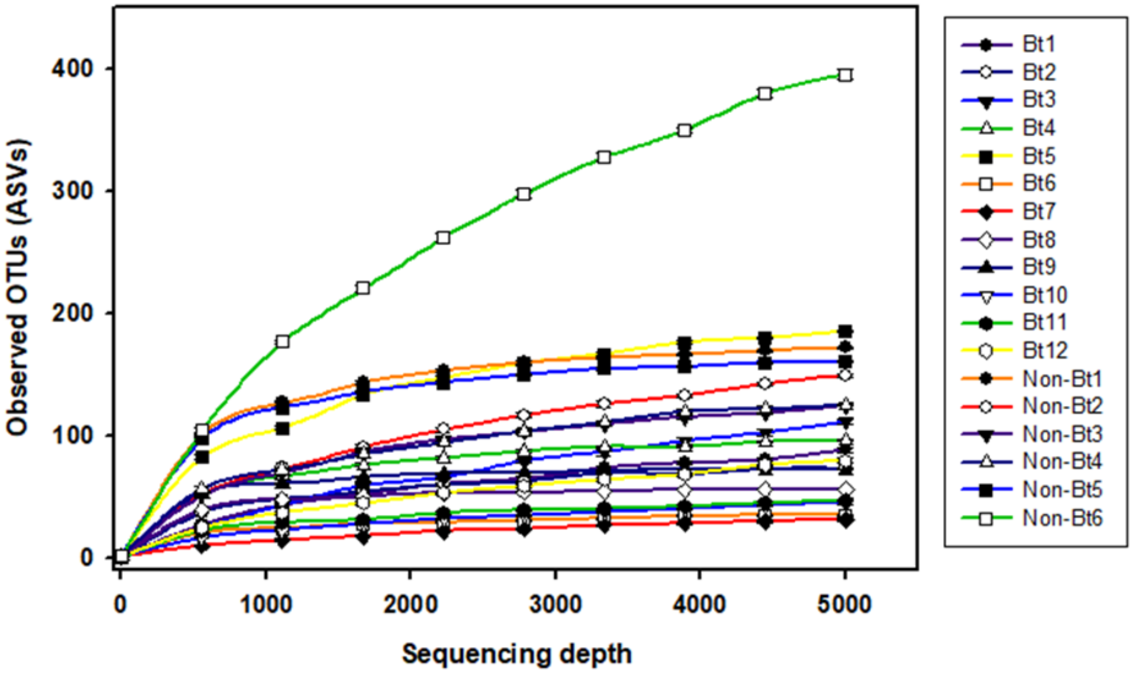

Figure S2. Rarefaction curves of the number of observed OTUs (sequence variants) from internal DNA samples of the cotton bollworm, *Helicoverpa zea*. Bt: sample originating from third stadium larvae from Bt (WideStrike, Cry1Ac + Cry1F) cotton. Non-Bt: sample originating from third stadium larvae from non-Bt cotton. Number after sample origin represents sample number. Sequences were obtained from amplified DNA fragments overlapping the V3–V4 hypervariable regions of the 16S rRNA gene.
